# The safety of an MRI simulation-guided boost after short-course preoperative radiotherapy for unresectable rectal cancer (SUNRISE): interim analysis of a randomized phase II trial

**DOI:** 10.1186/s13014-022-02182-4

**Published:** 2022-12-28

**Authors:** Wen-Yang Liu, Jin-Ming Shi, Ning Li, Xin Wang, Yuan-Hong Gao, Yihebali Chi, Yong-Kun Sun, Qing Zhao, Yong-Jian Zhu, Hong-Da Chen, Hui Fang, Ning-Ning Lu, Shu-Nan Qi, Bo Chen, Shu-Lian Wang, Yong-Wen Song, Yue-Ping Liu, Ye-Xiong Li, Zheng Liu, Hai-Tao Zhou, Jian-Wei Liang, Xi-Shan Wang, Hai-Zeng Zhang, Yuan Tang, Jing Jin

**Affiliations:** 1 Department of Radiation Oncology, National Cancer Center/National Clinical Research Center for Cancer/Cancer Hospital, Chinese Academy of Medical Sciences and Peking Union Medical College, Beijing, China; 2 Department of Radiation Oncology, West China Hospital, Sichuan University, Chengdu, China; 3 Department of Radiation Oncology, State Key Laboratory of Oncology in South China, Collaborative Innovation Center of Cancer Medicine, Sun Yat-sen University Cancer Center, Guangzhou, China; 4 Department of Medical Oncology, National Cancer Center/National Clinical Research Center for Cancer/Cancer Hospital, Chinese Academy of Medical Sciences and Peking Union Medical College, Beijing, China; 5 State Key Laboratory of Molecular Oncology, Department of Radiology, National Cancer Center/National Clinical Research Center for Cancer/Cancer Hospital, Chinese Academy of Medical Sciences and Peking Union Medical College, Beijing, China; 6 Medical Research Center, Peking Union Medical College Hospital, Chinese Academy of Medical Sciences and Peking Union Medical College, Beijing, China; 7 Department of Colorectal Surgery, National Cancer Center/National Clinical Research Center for Cancer/Cancer Hospital, Chinese Academy of Medical Sciences and Peking Union Medical College, Beijing, China; 8 Department of Radiation Oncology, National Cancer Center/National Clinical Research Center for Cancer/Cancer Hospital & Shenzhen Hospital, Chinese Academy of Medical Sciences and Peking Union Medical College, Shenzhen, 518116 China

**Keywords:** Rectal cancer, Preoperative, MRI, Radiotherapy boost

## Abstract

**Purpose:**

The safety of an MRI simulation-guided boost after short-course preoperative radiotherapy (SCPRT) for unresectable rectal cancer is assessed with a planned interim analysis.

**Methods and materials:**

Patients diagnosed with clinical stage T3-4 or regional lymph node-positive disease with positive mesorectal fascia or T4b disease evaluated by pelvic MRI were randomly assigned to the SCPRT-boost group (25 Gy in 5 fractions plus 4 Gy delivered to the gross tumor volume, followed by four cycles of chemotherapy) or preoperative chemoradiotherapy group (50 Gy in 25 fractions with concurrent chemotherapy). Then, patients received total mesorectal excision surgery after preoperative treatment. The primary endpoint was the R0 resection rate. The interim analysis was performed when 42 patients completed their assigned treatments.

**Results:**

From October 2018 to November 2019, a total of 43 patients were enrolled, and 42 patients were included in the interim analysis. During preoperative therapy, grade 3 or above toxicities were observed in 10/21 (47.6%) patients in the experimental group, and 4/21 (19.0%) patients in the control group. A total of 17 (81.0%) and 13 (61.9%) patients in the experimental group and control group underwent surgery, respectively. Overall, 65.1% of the patients achieved R0 resection in the intention-to-treat analysis. Surgery-related adverse complications were observed in 2 patients (11.8%) in the experimental group and 1 patient (7.7%) in the control group.

**Conclusion:**

Our results show that the toxicity of an MRI simulation-guided boost after SCPRT for unresectable rectal cancer is acceptable. Thus, this clinical trial will be continued as planned.

## Introduction

Colorectal cancer ranks third in incidence and fourth in mortality among commonly diagnosed cancer worldwide [1]. Approximately 70% of nonmetastatic rectal cancer cases are initially diagnosed as locally advanced rectal cancer (LARC), half of which were unresectable. Among them, except for those who can't tolerate surgery, the majority of patients with tumor unresectable were due to positive mesorectal fascia (MRF) evaluated by MRI or tumor invasion to adjacent organs [2]. For these patients, R0 resection remains the key strategy for curative treatment to ensure long-term survival [3]. Despite the adoption of the standard neoadjuvant treatment strategy recommended by the European Society for Medical Oncology (ESMO) guidelines [4], regardless of whether long-course preoperative chemoradiotherapy (CRT) or short-course radiotherapy followed by neoadjuvant chemotherapy is administered, an R0 resection rate of only approximately 70% can be achieved. Thus, the current conversion approaches are far from ideal [5].

For decades, different strategies, including simultaneous radiotherapy boost [6] or intensified neoadjuvant chemotherapy [5, 7], have been explored to improve the probability of achieving R0 resection in patients with unresectable LARC. Unfortunately, to date, no such approach has been demonstrated by randomized trials to improve the R0 resection rate compared to preoperative chemoradiotherapy (preCRT).

However, due to the critical role of R0 resection in the curative treatment of LARC, it is necessary to continue exploring new approaches to improve the R0 resection rate. Although recent reported RAPIDO trial [7] confirmed the disease-free survival benefit from total neoadjuvant treatment (TNT) in high-risk LARC, the percentage of patients included with positive MRF was only 60%, and unfortunately, clinical complete response or R0 resection was achieved in 85.7% from experimental group and 82.4% from control group. The question on optimal neoadjuvant strategy for MRF positive LARC remains open.

In previous studies, the results of the Stockholm III study show the superiority of hypofractionated radiation the pathological complete response (pCR) rate achieved by 5*5 short-course preoperative radiotherapy (SCPRT) with delayed surgery is higher than that achieved by conventional long-course radiotherapy alone) [8]. In contrast, although a better radiotherapy response may be achieved at an equivalent dose in 2-Gy fractions (EQD2) of 50–70 Gy according to the model proposed by Appelt et al. [9], another recent study reported that this dose range failed to improve pCR, even in the study arm, which received a conventional dose of 66.3 Gy EQD2 [10]. Theoretical advancements in the development of biological models [9] indicating the α/β ratio of rectal cancer estimated to be approximately 5 may account for the failures from boost study based on conventional fraction. Furthermore, in the Polish study, a trend toward an improved R0 resection rate was observed in the patients who received SCPRT combined with neoadjuvant chemotherapy (77% vs. 71%, *P* = 0.07) [5]. Similarly, better pCR and clinical complete response (cCR) rates were observed in the TNT group (22.5% vs. 12.6%, *P* = 0.001) in the STELLAR trial [11]. Both of the above trials highlight the contribution of SCRRT combined with an intensified neoadjuvant chemotherapy regimen to tumor regression.

Consequently, based on the evidence mentioned above and theoretical advances, we designed this multicenter randomized phase II trial to assess the safety and efficacy of an MRI simulation-guided boost after SCPRT for unresectable rectal cancer. Besides, according to a theory by Fowler et al. [12], a more biologically effective dose may result from hypofractionated radiotherapy. The hypofractionated boost model and neoadjuvant chemotherapy modality were used in this trial because of their promising therapeutic potential. Here, through a planned interim analysis of the first part of this prospective trial, we report the safety results.

## Methods and materials

This trial was a prospective multicenter randomized phase II trial and registered with ClinicalTrials.gov number NCT03714490. Ethics approval was obtained from the Ethics Committee of the Cancer Hospital, Chinese Academy of Medical Sciences. All patients signed informed consent forms before receiving treatment.

### Patient selection

Before enrollment, each patient was required to undergo the following examinations: colonoscopy, pathological biopsy, rectal MRI examination with small field and high-resolution T2 image for evaluating the invasion of MRF, chest-abdomen-pelvis enhanced CT, and electrocardiogram. Eligible patients met the following criteria: an age of 18 years or higher, Eastern Cooperative Oncology Group (ECOG) performance score ≤ 1, diagnosis of LARC with adenocarcinoma histology at clinical stage II/III (T3-4 or N + , AJCC 7th edition) with MRF positive or T4b disease as evaluated by pelvic MRI, the upper boundary of the tumor is below the peritoneal reflection, adequate blood counts and liver and kidney function, and no surgery, chemotherapy or other antitumor treatment after diagnosis. The exclusion criteria included the presence of distant metastases, recurrent rectal cancer, comorbid active Crohn's disease, ulcerative colitis or cancers other than basal cell carcinoma or in situ cervical carcinoma, and allergy to fluorouracil or platinum drugs.

### Treatment

**Fig. 1:**
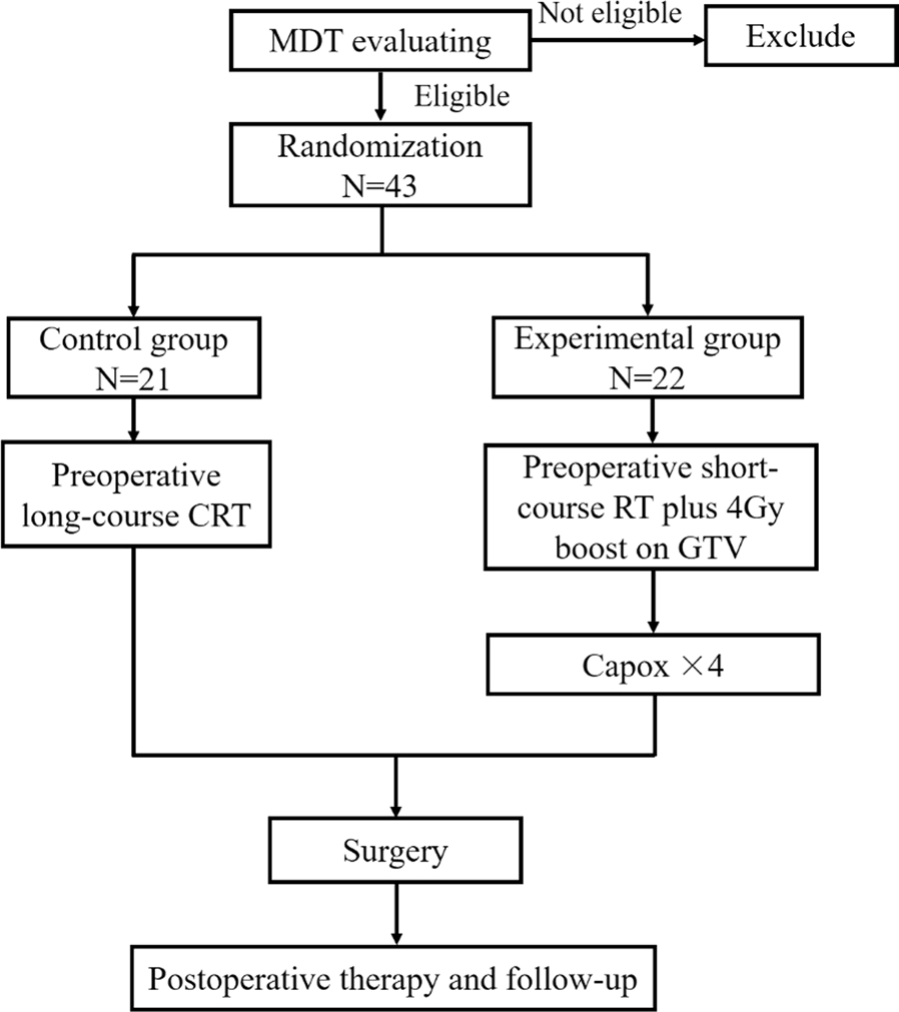
Study Flowchart (MDT: multiple disciplinary team; RT: radiotherapy; CRT: chemoradiotherapy)

Eligible patients were randomized into a control group and an experimental group. The study flow chart is presented in Fig. 1.

**Fig. 2:**
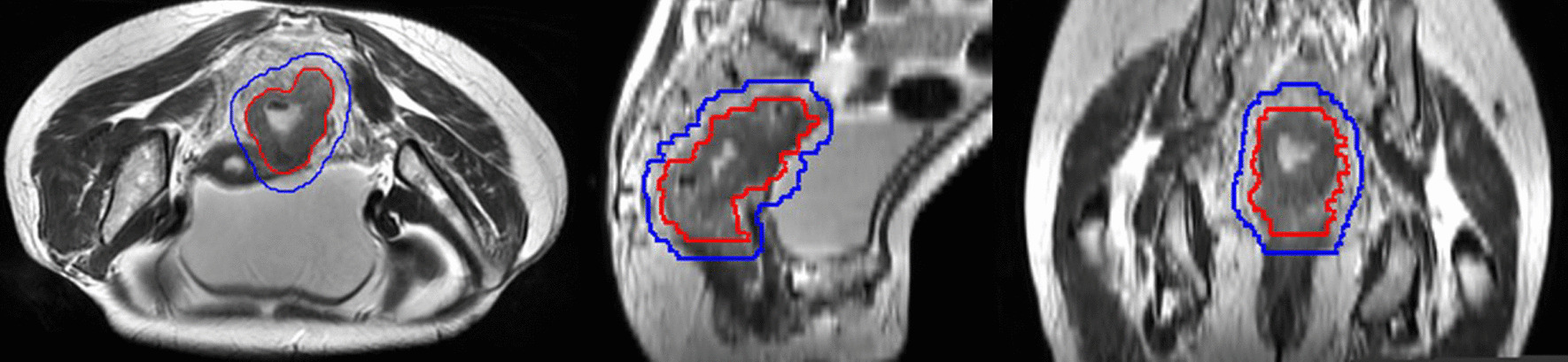
Example of target volume delineation of the boost area for the gross tumor on MRI simulation image (GTV: red, PGTV: blue)

The patients in the experimental group received SCPRT first, which consisted of 5 Gy × 5 delivered to the planning target volume (PTV) and an MRI simulation-guided 4 Gy boost dose delivered to the planning gross tumor volume (PGTV) (Fig. 2). One to two weeks after radiotherapy, four cycles of CAPOX regimens were administered (day 1: oxaliplatin 130 mg/m^2^ , day 1-14:  capecitabine 1000 mg/m^2^, bid, each cycle lasting for 21 days). One week after the preoperative therapy, patients received total mesorectal excision (TME) surgery and postoperative chemotherapy.

In the control group, patients received preCRT first (50 Gy in 25 fractions, concurrent with capecitabine at a dose of 825 mg/m2 twice daily on days of radiotherapy). Four to six weeks after CRT, TME surgery and postoperative chemotherapy were administered.

For all enrolled patients, both CT and high-resolution MRI simulation were applied before the treatment. What’s more, after five fractions of radiotherapy in the experimental group, a second MRI simulation was conducted for the gross tumor volume (GTV) boost. MRI-CT fusion was used to define the target volume. The GTV was defined as the primary tumor determined by MRI and CT enhanced simulation images and colonoscopy. The clinical target volume (CTV) contained the GTV, mesorectum and high-risk regional lymph nodes. The PTV and PGTV were derived from the CTV and GTV plus 1 cm in the cranio-caudal direction and 0.7 cm in the anterior-posterior and left-right directions [13]. Volumetric modulated arc therapy (VMAT) was recommended during radiotherapy. Delineation of the primary organs at risk (OARs) was determined according to the RTOG guidelines [14] and the dose limitations were as follows: in the experimental group, V25 femur head < 5%, V25 bladder < 50%, V25 colon < 10%, Dmax colon < 27 Gy, V25 small bowel < 10%, and Dmax small bowel < 27 Gy; in the control group, V50 femur head < 5%, V50 bladder < 50%, V50 colon < 20%, Dmax colon < 60 Gy, V50 small bowel < 20%, and Dmax small bowel < 55 Gy [13, 15]. During the IGRT treatment, patients in the experimental group received cone beam computed tomography (CBCT) scans before each fraction. In the control group, patients received CBCT scans daily in the first week and then once a week during radiotherapy. There were no limitations in terms of the choice of postoperative chemotherapy regimens for enrolled patients in either group.

### Tumor response evaluation and toxicity criteria

Patients underwent routine blood and biochemical testing weekly during the preoperative radiotherapy and chemotherapy period. Four weeks after radiotherapy and before surgery, CEA and CA 199 levels were measured and pelvic MRI, colonoscopy, and chest-abdomen-pelvis enhanced CT were performed. These tests were repeated every three months during the first 2 years, every six months for 3–5 years, and once a year thereafter. The Common Terminology Criteria of Adverse Events (CTCAE) version 4.0 was used to assess the toxicities that occurred during therapy. The Late Effects Normal Tissue Task Force-Subjective, Objective, Management and Analytic (LENT-SOMA) scoring systems was adopted to evaluate the long-term toxicity [16]. Tumor regression grading was reported based on the classification system proposed by Dworak et al. [17]. Radiotherapy or chemotherapy was suspended if grade 3 toxicities occurred except for perianal skin reaction. After symptomatic treatment, therapy was resumed when the grade of adverse effects was reduced to 0–1.

### Objectives

The primary endpoint of this trial was to compare the R0 resection rate (defined as the negative microscopic resection margin) between the two groups. The presence of cancer cells within 1 mm of the surgical margin was defined as R1 resection. The secondary endpoints were acute or late toxicities (occurring 30 days after surgery), the pathological complete response (pCR) rate, disease-free survival (DFS: the time from surgery to recurrence or death), overall survival (OS: the time from diagnosis to death), local recurrence-free survival (LRFS: the time from surgery to local recurrence), distant metastasis-free survival (DMFS: the time from diagnosis to metastasis), and health related quality of life (QoL).Moreover, we collected patient blood and tissue samples to explore biomarkers for predicting the treatment response and survival outcomes.

### Sample size consideration and interim analysis design

According to the historical data at our hospital and the results of the phase III clinical trial in Poland [5, 11], we assumed that the R0 resection rate in the control group would be 65%. Taking into consideration that there was a 3% drop-out rate and 4% of patients refused surgery, a total of 200 cases would need to be included in this study, in order to ensure that a one-sided test with α =0.05 has 80% power to detect a 16% difference in R0 resection rate between two groups.

We used the 23% difference in the incidence of serious toxicities between the study group and control group reported by the phase II safety results from STELLAR as a reference, so interim safety assessment was planned when twenty-one patients in each group completed neoadjuvant therapy. The hypothesis is that if the difference in the incidence of grade 3–5 toxicities between the study group and control group exceeds 35%, with an α level of 0.05 and a power of 0.9 (one-sided) by the Z pooled test, the safety of the new approach would be deemed unacceptable, and hence, the investigator would need to modify the study protocol or terminate the study. The interim analysis also included perioperative complications as well as short-term outcomes such as pCR.

Stratified blocked randomization scheme by T4b status was generated by the statistical software R using predefined seed. The allocation scheme was only revealed after successful registration for each participant. The staff performing the randomization was not involved in participants recruitment.

### The calculation of biological effective doses

We used the time-corrected linear square formula proposed by Fowler et al. [12] and parameters from Colorectal Cancer Collaborative Group [18] to calculate the biological effective dose. BED = *n* × d(1 + [d/α/β]) − α/γ × (T − Tk), *n* is the number of fraction, d is the dose of each fraction, α/β is 5, α/γ = 0.6 Gy, T = total days of treatment, and the proliferation delay Tk was 7. According to this formula, the BED was 56.6 Gy and 53.2 Gy in the experimental group and control group. EQD2 for the experimental radiation dose fraction is 40.9.

### Statistical analyses

To avoid underestimating toxicity, a safety evaluation was performed using a per-protocol analysis. The preliminary efficacy analyses were performed with the intention-to-treat population. Descriptive statistics were used for qualitative and quantitative variables. T-test was used to compare quantitative variables between groups in the Table 1. The chi-square test or Fisher's exact test was used to compare categorical data between groups in the Table 1. All analyses were conducted using SPSS Statistics 22.0. The level of significance was set to *P* < 0.05.

**Table 1:** Baseline patient characteristics

Characteristics	Experimental group No. (%)	Control group No. (%)	*P*
Total No. of patient	21	21	
*Age(years)*			
Median (range)	52 (31–66)	62 (48–65)	0.024
*Sex*			0.026
Female	11 (52.4)	4 (19.0)	
Male	10 (47.6)	17 (81.0)	
*EMVI status*			0.500
Positive	14 (66.7)	15 (71.4)	
Negative	7 (33.3)	6 (28.6)	
*MRF status*			1.000
Positive (cT3-cT4a)	18 (85.7)	18 (85.7)	
Positive (cT4b)	3 (14.3)	3 (14.3)	
Negative (cT3-cT4a)	0 (0)	0 (0)	
Negative (cT4b)	0 (0)	0 (0)	
*MRI T stage*			0.371
cT3	18 (85.7)	15 (71.4)	
cT4a	0 (0)	3 (14.3)	
cT4b	3 (14.3)	3 (14.3)	
*MRI N stage*			0.722
cN0	1 (4.8)	3 (14.3)	
cN1	6 (28.6)	6 (28.6)	
cN2	14 (66.7)	12 (57.1)	
*Clinical stage*			0.226
II	0 (0)	3 (1.4)	
IIIA	0 (0)	0 (1.4)	
IIIB	18 (85.7)	13 (61.9)	
IIIC	3 (1.4)	5 (23.8)	
*Tumor location*			1.000
Lower	18 (85.7)	19 (90.5)	
Middle	3 (14.3)	2 (9.5)	

*EMVI* extramural vascular invasion; *MRF* mesorectal fascia

## Results

For the interim analysis, 43 patients were enrolled from October 2018 to November 2019. Among them, one patient in the experimental group refused to receive the distributed treatment and, therefore, was not enrolled in the safety analysis. A total of 21 patients each in the experimental group and control group ultimately received the assigned treatments. As of October 2020, the median follow-up time was 15.8 months (range: 8.1–24.0 months).

### Patient characteristics

The baseline characteristics of the two groups are listed in Table 1. No significant differences in the clinical features were observed between these two groups, including the extramural vascular invasion (EMVI) status, tumor location and clinical stage (*P* > 0.05), except for the median age (*P* = 0.024) and sex (*P* = 0.026).

### Treatment toxicities and compliance

One patient in the experimental group refused the last fraction and ultimately received a radiation dose of 25 Gy. All other patients completed the entire neoadjuvant CRT treatment schedule without interrupt as planned. Grade 3 or higher toxicity was observed in 10/21 (47.6%) patients in the experimental group and 4/21 (19.0%) patients in the control group. The most common serious acute toxicities were proctitis (28.6%), pain (23.8%), dermatitis (9.5%), leukopenia (9.5%), and diarrhea (9.5%) in the experimental group, and diarrhea (9.5%) in the control group. The difference in the incidence of severe toxicity between the two groups was 28.6% (*P* = 0.050 for one-sided and 0.100 for two-sided by Fisher’s exact test). All treatment-related toxicities that occurred during preoperative treatment are listed in Table 2.

**Table 2:** Acute toxicities that occurred during preoperative treatment

Toxicities	Experimental group *N* = 21 (%)				Control group *N* = 21 (%)	
	SCPRT		NCT		NCRT	
	Grade 1–2	Grade 3–4	Grade 1–2	Grade 3–4	Grade 1–2	Grade 3–4
*Hematological*						
Total	6 (28.6)	0	14 (66.7)	2 (9.5)	10 (47.6)	1 (4.8)
Leukopenia	6 (28.6)	0	14 (66.7)	1 (4.8)	10 (47.6)	1 (4.8)
Thrombocytopenia	0	0	0	1 (4.8)	0	0
Neutropenia	0	0	3 (14.3)	0	1 (4.8)	0
Anemia	2 (9.5)	0	2 (9.5)	0	3 (14.3)	0
*Non-Hematological*						
Total	12 (57.1)	4 (19.0)	14 (66.7)	2 (9.5)	16 (76.2)	3 (14.3)
Fatigue	3 (14.3)	0	3 (14.3)	0	7 (33.3)	0
Bleeding	6 (28.6)	0	2 (9.5)	0	7 (33.3)	0
Diarrhea	7 (33.3)	2 (9.5)	1 (4.8)	0	4 (19.0)	3 (14.3)
Abdominal distension	1 (4.8)	1 (4.8)	1 (4.8)	0	0	0
Anorexia	2 (9.5)	0	10 (47.6)	0	1 (4.8)	0
Nausea	3 (14.3)	0	10 (47.6)	1 (4.8)	2 (9.5)	0
Vomiting	1 (4.8)	0	7 (33.3)	1 (4.8)	0	0
Pain	8 (38.1)	4 (19.0)	3 (14.3)	1 (4.8)	13 (61.9)	1 (4.8)
Dermatitis	7 (33.3)	1 (4.8)	1 (4.8)	0	9 (42.9)	1 (4.8)

*SCPRT* short-course preoperative radiotherapy; *NCT* neoadjuvant chemotherapy; *NCRT* neoadjuvant chemoradiotherapy

### Surgical results and tumor pathological response

Finally, 17(81.0%) patients in the experimental group and 13(61.9%) patients in the control group underwent surgery. In the experimental group, four patients did not undergo surgery for the following reasons: the tumor was deemed unresectable (1), lung metastasis occurred prior to surgery (1) and a watch-and-wait strategy was chosen due to cCR (2). In the control group, eight patients did not receive surgery due to refusal (2), an unresectable tumor (1), and metastasis occurring prior to surgery (5).

In the intention-to-treat (ITT) analysis, 15 (68.2%) patients in the experimental group and 13 (61.9%) patients in the control group achieved R0 resection. Among the 30 patients who underwent surgery, the pCR rate was observed in 5 (16.7%) patients. Dworak tumor regression grades 2–4 were observed in 24 (80%) patients. Surgery-related adverse complications were observed in two patients in the experimental group (rectovaginal fistula in one patient and intestinal obstruction in the other patient) and in one patient in the control group (delayed wound healing). The details of the surgical and tumor pathological responses are presented in Table 3.

**Table 3:** Surgical and tumor pathological response

	Total *N* = 30 (%)
*Type of surgery*	
Miles	19 (63.3)
Dixon	11 (36.7)
R0 resection rate	28 (93.3)
pCR rate	5 (16.7)
Number of lymph nodes dissected (Median, range)	15 (3–30)
Number of lymph nodes positive (Median, range)	0 (0–9)
*Distance to CRM* *(Median, range mm)*	
≤ 1 mm	2 (6.7)
> 1 mm	28 (93.3)
*Pathologic T stage*	
T0	5 (16.7)
T1	2 (6.7)
T2	9 (30.0)
T3	12 (40.0)
T4	2 (6.7)
*Pathologic N stage*	
N0	16 (53.3)
N1	10 (33.3)
N2	4 (13.3)
*Tumor regression*	
Grade 1	3 (10.0)
Grade 2	14 (46.7)
Grade 3	5 (16.7)
Grade 4	5 (16.7)
unknown	3 (10.0)

*pCR* pathological complete response; *CRM* circumferential resection margin

## Discussion

R0 resection is imperative to achieving local control to confer survival benefit in rectal cancer patients [19], especially those with initially unresectable tumors. Much room for improvement still exists in the conversion efficacy of recommended preCRT or TNT [4, 20]; nevertheless, there is no robust evidence supporting any treatment strategy that can improve the R0 resection rate compared to preCRT.

Paradoxically, although several radiotherapy dose-escalation studies have been conducted in the field of preoperative chemoradiotherapy for LARC [6, 10, 21, 22], most of them aimed to achieve a higher pCR rate, while few have focused on the conversion effect on R0 excision. In addition, due to the limitations of previous techniques, MRI simulation technology has not yet been adopted. As a result, it is difficult to accurately delineate GTV on CT simulation images, and the whole mesorectal region boost must be incorporated into the study design. Regrettably, there is no clear evidence that the pCR rate can be significantly improved with a conventional fraction boost.

To improve the R0 resection rate, as a first step, we designed this phase II randomized trial using an MRI simulation-guided boost technique. Here, we report the safety results in the form of an interim analysis. For the first time, SCPRT with a 4 Gy boost and sequential neoadjuvant chemotherapy was shown to be feasible and safe in the current study.

Boost after SCPRT was used in the trial design in conjunction with current advances in radiobiology theory and clinical data [5, 8, 11]. Regarding the development of biological models for LARC [9], the α/β ratio of rectal cancer has been estimated to be approximately 5, indicating that these patients may benefit more from hypofractionated radiation doses in tumor regression. In addition, based on a theory by Fowler et al. [12], hypofractionated radiotherapy may have a more biologically effective dose, given the overall treatment time and risk of tumor repopulation.

In an earlier study reported by Bujko et al. [5], tumor fixation was included in the unresectable criteria, and a trend toward R0 resection improvement was observed with SCPRT combined with neoadjuvant chemotherapy. As MRF status can well predict the R0 resection probability, it was included as a standard for inclusion in this study, which may help clarify this issue further [23]. Similarly, in the STELLAR trial, more tumor shrinkage was found in the TNT group [11]. Hence, four cycles of neoadjuvant chemotherapy combined with SCPRT were employed in the current trial due to its conversion potency. Regarding safety, almost the same serious toxicity rate was observed in the current experimental arm as that observed in intensified neoadjuvant treatment studies, including the RAPIDO (48%) [7] and PRODIGE23 (45%) trials [24]. Despite numerically higher grade 3 or above toxicities occurring in this experimental group than in the STELLAR trial [11], this is reasonable because all patients were MRF positive, indicating more aggressive gross tumors, as the control arm of this study also experienced more toxicities. Thus, we believe that the use of an MRI simulation-guided boost after SCPRT for LARC is safe and feasible.

On the other hand, although postoperative complications may be a concern for boost studies, the grade 3–5 surgical adverse events (11.8%, one rectovaginal fistula and one intestinal obstruction) observed in the experimental group were similar to those observed in the STELLAR [11] (14–15.7%) and RAPIDO (14–15%) [25] trials. Although long-term toxicity from hypofractionated radiotherapy deserves further attention, the increased possibility of R0 resection may outweigh the risk of injury.

In most previous studies, conformal radiotherapy and CT simulation were employed (CT + 3D CRT) [6, 22] or (CT + IMRT) [21, 22]. The MRI simulation-guided boost delivered to the GTV in the current study would make the boost field smaller and delineation more precise, as has been reported before [22]. To reduce the possible impact of variability during radiotherapy on precise boost delivery, MRI-simulation was repeated before delivering the boost to confirm the location of the GTV.

It is not yet time for an efficacy comparison of the primary endpoint, as this is an interim analysis. However, 68.2% and 61.9% of the patients achieved R0 resection in the experimental group and control group respectively, which is comparable to the R0 resection rate in the control group of the Polish II trial [5] and our historical data. Hence, there is reason to believe that this trial should be continued.

As an interim report and safety analysis, this study has certain limitations. Firstly, with the rapid development of adaptive radiotherapy during the study, the patients who received boost during short-course radiotherapy may benefit more from the MRI-Linac [26], which can provide online treatment MRI images and allow oncologists to precisely target tumors, as shown in the design of the THUNDER-2 study [27]. Secondly, some patients did not undergo surgery due to cCR, which could cause difficulties for the primary endpoint evaluation in the future. Thus, there is a need for reasonable revisions to the primary endpoint.

## Conclusion

In summary, the toxicities of short-course preoperative radiotherapy delivered to the planning target volume and a 4 Gy MRI simulation-guided boost delivered to the planning gross tumor volume for unresectable locally advanced rectal cancer are acceptable. Continued enrollment in this clinical trial should be completed as planned.

## Data Availability

Research data are stored in an institutional repository and will be shared upon request to the corresponding author.

## Abbreviations

cCR: Clinical complete response
CRT: Chemoradiotherapy
CTV: Clinical target volume
CBCT: Cone beam computed tomography
DMFS: Distant metastasis-free survival
LARC: Locally advanced rectal cancer
LRFS: Local recurrence-free survival
MRF: Mesorectal fascia
OARs: Organs at risk
OS: Overall survival
PTV: Planning target volume
PGTV: Planning gross tumor volume
GTV: Gross tumor volume
pCR: Pathological complete response
preCRT: Preoperative chemoradiotherapy
QoL: Quality of life
VMAT: Volumetric modulated arc therapy
SCPRT: Short-course preoperative radiotherapy
TNT: Total neoadjuvant treatment
TME: Total mesorectal excision
